# Maternal feeding practices in relation to dietary intakes and BMI in 5 year-olds in a multi-ethnic Asian population

**DOI:** 10.1371/journal.pone.0203045

**Published:** 2018-09-18

**Authors:** Phaik Ling Quah, Ginanjar Syuhada, Lisa R. Fries, Mei Jun Chan, Hui Xian Lim, Jia Ying Toh, Ray Sugianto, Izzuddin M. Aris, Yung Seng Lee, Fabian Yap, Keith M. Godfrey, Peter D. Gluckman, Yap- Seng Chong, Lynette P. Shek, Kok Hian Tan, Ciaran G. Forde, Mary F. F. Chong

**Affiliations:** 1 Singapore Institute for Clinical Sciences, Agency for Science, Technology, and Research, Singapore, Singapore; 2 Nestle Research Center, Lausanne, Switzerland; 3 Saw Swee Hock School of Public Health, National University of Singapore, Singapore, Singapore; 4 Department of Pediatrics, Yong Loo Lin School of Medicine, National University of Singapore and National University Health System, Singapore, Singapore; 5 Khoo Teck Puat-National University Children’s Medical Institute, National University Health System, Singapore, Singapore; 6 Pediatric Endocrinology, KK Women’s and Children’s Hospital, Singapore, Singapore; 7 Duke-NUS Graduate Medical School, Lee Kong Chian School of Medicine, Singapore, Singapore; 8 Lee Kong Chian School of Medicine, Nanyang Technological University, Singapore, Singapore; 9 Medical Research Council Lifecourse Epidemiology Unit and National Institute for Health Research Southampton Biomedical Research Centre, University of Southampton and University Hospital, Southampton National Health Service Foundation Trust, Southampton, United Kingdom; 10 Liggins Institute, University of Auckland, Auckland, New Zealand; 11 Department of Obstetrics & Gynaecology, Yong Loo Lin School of Medicine, National University of Singapore and National University Health System, Singapore, Singapore; 12 Department of Obstetrics and Gynaecology, KK Women’s and Children’s Hospital, Singapore, Singapore; 13 Clinical Nutrition Research Center, Singapore Institute for Clinical Sciences (SICS), Agency for Science, Technology and Research (A*STAR), Singapore, Singapore; 14 Department of Physiology, Yong Loo Lin School of Medicine, National University of Singapore, Singapore; Medical University of Vienna, AUSTRIA

## Abstract

**Background:**

In Asia, little is known about how maternal feeding practices are associated with dietary intakes and body mass index (BMI) in preschoolers.

**Objective:**

To assess the relationships between maternal feeding practices with dietary intakes and BMI in preschoolers in Asia using cross-sectional analysis in the GUSTO (Growing Up in Singapore Towards healthy Outcomes) cohort.

**Participant settings:**

Mothers (n = 511) who completed the Comprehensive Feeding Practices Questionnaire (CFPQ) and a semi-quantitative Food Frequency Questionnaire (FFQ) when children were 5 years old.

**Statistical analysis:**

Associations between 12 maternal feeding practices (mean scores divided into tertiles) and children’s dietary intakes of seven food groups and BMI z-scores were examined using the general linear regression model. Weight and height of the child were measured, and dietary intakes derived from the FFQ.

**Results:**

Compared to those in the low tertile, mothers in the high tertile of modelling healthy food intakes had children with higher intakes of vegetables[+20.0g/day (95%CI:11.6,29.5)] and wholegrains[+ 20.9g/day (9.67,31.1)] but lower intakes of sweet snacks[-10.1g/day (-16.3,-4.94)] and fast-foods[-5.84g/day (-10.2,-1.48)]. Conversely, children of mothers in the high tertile for allowing child control (lack of parental control) had lower intake of vegetables[-15.2g/day (-26.6,-5.21)] and wholegrains[-13.6g/day (-22.9,-5.27)], but higher intakes of sweet snacks[+13.7g/day (7.7, 19.8)] and fast-foods[+6.63g/day (3.55,9.72)]. In relation to BMI at 5 years, food restrictions for weight was associated with higher BMI z-scores [0.86SD (0.61,1.21)], while use of pressure was associated with lower BMI z-scores[-0.49SD(-0.78,-0.21)].

**Conclusions and implications:**

Modelling healthy food intakes by mothers was the key feeding practice associated with higher intakes of healthy foods and lower intakes of discretionary foods. The converse was true for allowing child control. Only food restrictions for weight and use of pressure were associated with BMI z-scores.

## Introduction

Food preferences formed in childhood can track to adulthood[1], and as interventions studies have shown, parental control of the food environment at home using a variety of feeding practices was able to influence their child’s dietary intake[2,3].

Feeding practices are defined as parental-child feeding interactions which determine how, when and why children are fed [4], and may be potentially modifiable determinants of a child’s weight status through dietary intakes. Identifying beneficial ones [5,6] will help inform future intervention studies aiming to improve the overall diet quality in children, and promote healthy weight gain. Feeding practices that promote healthy eating habits in accordance to worldwide dietary guidelines[7] which recommend low fat and sugar consumption [8,9], and higher fruits, vegetables and wholegrains consumption are of primary interest [10,11].

Systematic reviews have shown that studies examining maternal feeding practices in relation to children’s dietary intake and BMI have largely focused on feeding practices such as restriction of food intakes, the use of pressure to eat, and monitoring of unhealthy food intakes in children[12–14]. More recently, an increasing number of studies have examined feeding practices using the Comprehensive Feeding Practice Questionnaire (CFPQ), which in total, captures 12 feeding practices [15–18]. Validated in parents of young children (2–8 years old) [19], this questionnaire is an extension of previous measures of feeding practices [4,20], and represents a more complete range of positive and negative feeding practices that may influence child dietary intakes and BMI [15,16,18,21].

However, it is noted that some of these previous studies were focused on preschoolers of particular weight statuses (e.g. overweight)[18], and those from low-socioeconomic (SES) backgrounds[21], thus potentially limiting the generalizability of these findings. Additionally, many studies focused on primarily fruit and vegetables intakes [17,18], leaving other aspects of the diet less explored. Furthermore, few studies have examined the influence of these feeding practices on both dietary intakes and BMI [21]. Some studies [15,16,21] conducted only univariate analysis without including other variables that could potentially confound the association studied, for example, maternal age or educational levels which have been previously shown to be associated with both maternal feeding practices[22,23], as well as dietary intakes of children [24,25]. Thus, the lack of accounting for confounding factors may result in inaccurate conclusions from these previous findings [15,16,21].

At present, studies which use measures of maternal feeding practices from the CFPQ are typically from Caucasian and other non-Asian populations [15,17,18,21]. Asian feeding practices differ from those of other ethnicities(Whites, Blacks and Hispanics)[26], hence understanding the influence of maternal feeding practices on children’s dietary intakes in an Asian population can help inform the development of more culturally appropriate interventions. Furthermore, it can guide parental feeding practices in a way that maximizes the positive influences, and minimizes the development of unhealthy feeding behaviors [2,3].

We aim to explore the 12 maternal feeding practices captured in the CFPQ and their relationships to the dietary intakes and BMI of preschoolers in Singapore. We hypothesize that positive maternal feeding practices such as monitoring (of unhealthy food intakes), modelling (of healthy food intakes), encouraging balance and variety in children’s diet, promoting a healthy home environment (by making healthy food available at home), teaching children about nutrition and child involvement (in meal preparation) will be associated with healthier diets such as higher fruit, vegetable and wholegrain intakes and a lower BMI. By contrast, we hypothesize that negative feeding practices such as food restrictions for health and weight, use of pressure, food as reward, food for emotional regulation and child control will be associated with less healthy diets such as higher intakes of sugar sweetened beverages, sweet snacks, fast foods, and fried foods, and a higher BMI.

## Methods

### Study design and participants

Data for this study were collected within the Growing Up in Singapore Toward healthy Outcomes (GUSTO) Study (www.clinicaltrials.gov, NCT01174875). Detailed information on study design and measurements has been previously published[27]. In this study, pregnant Chinese, Malay, and Indian women were recruited at 14 weeks of gestation from 2 major public maternity units of Kandang Kerbau Women’s and Children’s Hospital (KKH) and the National University Hospital (NUH) in Singapore from June 2009 to September 2010. To be eligible, participants had to be Singaporean citizens or permanent residents, of Chinese, Malay or Indian ethnicity with parents of homogeneous ethnic background, have the intention to deliver in NUH or KKH, plan to reside in Singapore in the upcoming 5 years, and had to be willing to donate birth tissue at delivery (cord, placenta and cord blood). The major exclusion criterion was having a serious pre-pregnancy health condition such as type 1 diabetes. The study protocol was approved by the National Health Care Group Domain-Specific Review Board and SingHealth Centralized Institutional Review Board, and all participants gave written informed consent before recruitment.

Of the original 1247 participants enrolled in the study, 511 completed the CFPQ and FFQ at 5 years of age. In total, 736 mother-child pairs were excluded from the study, of whom 95 underwent in vitro fertilization or had twins, 140 dropped out for personal reasons, and 461 did not fully complete the questionnaire, 40 did not have FFQ data.

### Measures

#### Assessment of maternal feeding practices at year 5

The Comprehensive Feeding Practices Questionnaire (CFPQ) [28] was administered to the mothers in English which is the administrative language in Singapore. The CFPQ comprises of 49 items which were answered using two response formats depending on whether the items addressed frequency or degree. The response formats were ‘‘never, rarely, sometimes, mostly, always” or ‘‘disagree, slightly disagree, neutral, slightly agree, agree” [19]. Encourage balance and variety (4 items, e.g., ‘I encourage my child to try new foods’), Environment (4 items, e.g., ‘Most of the food I keep in the house is healthy’), Involvement (3 items, e.g., ‘I involve my child in planning family meals’), Modelling (4 items, e.g., ‘I model healthy eating for my child by eating healthy foods myself’), Monitoring (4 items, e.g., ‘How much do you keep track the sweets that your child eats?’), Teaching about nutrition (3 items, e.g., ‘I discuss with my child the nutritional value of foods’), Child control (5 items, e.g., ‘Do you let your child eat whatever s/he wants?’), Emotion regulation (3 items, e.g., ‘When the child gets fussy, is giving him/her something to eat or drink the first thing you do?’), Food as reward (3 items, e.g., ‘I offer my child his/her favourite foods in exchange for good behaviour’), Pressure (4 items, e.g., ‘My child should always eat all of the food on his/her plate’), Restriction for health (4 items, e.g., ‘I have to be sure that my child does not eat too much of his/her favourite foods’), and Restriction for weight control (8 items, e.g., ‘I encourage my child to eat less so he/she won’t get fat’) [28]. Mean scores were calculated from all the items of each subscale only when the questionnaire was fully completed without any missing data. Higher scores on a subscale indicated greater use of the particular feeding practice. The questionnaire has been validated among parents of children aged 18 months to 8 years [28], and suitable for use in this age group.

#### Maternal and child characteristics

Data on maternal ethnicity and educational level were collected from participants at the recruitment visit. Measured weight at ≤14 weeks gestation was extracted retrospectively from clinic records, and was used to calculate and represent pre-pregnancy BMI. Mothers’ height was measured at 26–28 weeks of gestation using a stadiometer (SECA206, Hamburg, Germany). The BMI at ≤14 weeks gestation showed a high correlation with pre-pregnancy BMI (r = 0.965). Information on child sex and birth order was extracted from obstetric records. Frequency and duration of child milk feeding practices was collected at 3, 6, 9, and 12 months of age with the use of interviewer-administered questionnaires to estimate the duration of exclusive breastfeeding. The weight of the child at 5 years of age was obtained using with the use of a calibrated digital scale (SECA model 813; SECA Corp.) to the nearest 10 g. Standing height was measured with the use of a stadiometer (SECA model 213). For reliability, all measurements were taken in duplicates and averaged. Child BMI was calculated as weight divided by the square of height. Based on WHO Child Growth Standards 2006, age and sex-adjusted BMI z-scores were derived using WHO Anthro software (Version 3.2.2) [29].

#### Assessment of dietary intake at 5 years of age

The participants were asked to report their child’s food and beverage intake through a Food Frequency Questionnaire (FFQ) for our Singapore population when the child turned 5 years old (validation paper under review)[30]. In brief, the FFQ is quantitative, and contains 112 food items that capture the child’s habitual intake for the month prior to the questionnaire administration, and from the child’s dietary intake total energy intake could be derived. The FFQ was interviewer administered by trained staff. Caregivers (e.g. mothers, fathers, grandparents or nannies) indicated the frequency of consumption of particular items on a variety of time scales (per month, week, or day). To assist with portion estimation, pictures were provided of food with various portion sizes based on typical household units (eg: slices, glasses, cups, plates, pieces, spoons and teaspoons). Dietary intakes of food were standardized to daily frequencies, and multiplied by average amount per serving in grams (g), to obtain total intake in grams per day (g/day). Similarly, the consumption of beverages was standardized to daily frequencies, and multiplied by the average amount per serving in milliliters (mLs) to obtain total intake in milliliters per day (mL/day).

For analysis, seven food-groups were created by combining items from the FFQ together: fruits (e.g. apple, banana), vegetables (e.g. carrot, pumpkins, corn), wholegrains (e.g. wholemeal bread, multigrain bread, soft wholegrain bread), sugar-sweetened beverages (SSBs; e.g. fruit drinks, soft drinks), sweet discretionary snacks (e.g. sweets, ice cream), fast-foods (e.g. non-core convenience foods such as nuggets, pizza), and deep-fried foods (e.g. fried chicken, fried beef/lamb, fried fish, fried potatoes).

### Statistical analyses

Pearson correlations were used to analyse the associations among the 12 CFPQ subscales. The strengths of the correlations were interpreted based on the absolute value of *r* as very weak (*r* = 0.00–0.19), weak (*r* = 0.20–0.39), moderate (*r* = 0.40–0.59), strong (*r* = 0.60–0.79) and very strong (*r* = 0.80–1.00) [31].

Based on the mean scores for each subscale in the CFPQ, mothers were divided into tertile categories of high, medium and low scores of equal sample size using Visual Binning with equal percentiles in SPSS® for more intuitive interpretation of the results. To examine the association between the 12 maternal feeding practice subscales with children’s dietary intake and BMI, the Generalized Linear Model with Gamma/Inverse Gaussian distribution and identity link was used. This method was applied to handle the dietary intake data that was mostly positively skewed in this study [32,33]. Since this method is recommended only for non-zero positively skewed data, a constant value of 10g/day was added to the entire dataset to account for variables which contained zeros [34]. Altogether, this method handles excess zeroes and skewness simultaneously without having to transform FFQ dietary intake values [33].

We presented models adjusting for confounding variables, such as maternal ethnicity, maternal education level, maternal pregnancy BMI at ≤14 weeks gestation, child sex, child’s birth order and breastfeeding duration. These confounders were chosen for this analysis based on the associations with the exposure of maternal feeding practices [22], and outcomes of dietary intake and BMI[25,35] as previously reported from our studies using the GUSTO cohort. The graphical presentation of confounding in a directed acyclic graph(DAG) is available in S1 Fig.

All the selected confounders contributed to more than a 5% effect estimate change in the adjusted models compared to the unadjusted models.

The beta coefficient values were then interpreted as the mean difference in dietary intake between the high or medium groups with the lowest tertile group as the reference group. A positive difference indicates greater intake compared to the reference group, while a negative difference indicates lower intake compared to the reference group.

Frequencies of missing demographic data were low (<5%), and were only for educational level (n = 4) and family income (n = 31), and were assumed to be missing at random. To account for covariates with missing data, missing values were imputed 20 times using multiple imputation analysis, and the results of the 20 datasets were pooled[36].

All analyses were performed by using SPSS® software version 23.0 (IBM). Statistical significance in regression models was identified by a *p* value of <0.006, determined by applying the Bonferroni corrections accounting for the maximum eight outcome variables examined in this study, and to minimize type I errors due to multiple comparisons.

## Results

### Study sample characteristics

The 511 participants in this study had mothers with mean age of 30 years, and about half (47%) were of Chinese ethnicity. About 70% of the mothers have obtained post-secondary education, and 57.1% have an average household income of S$2000–5999 per month. Half of the children are male (52.1%) and 57.5% were not first-born (Table 1). The S1 Table shows that there were no significant differences in the maternal and infant characteristics between the participants and the non-participants, with the notable exception of ethnicity, where the responder group had a higher percentage of Malays and Indians.

**Table 1:** Characteristics of included GUSTO study participants (n = 511) ^1^.

	Study participants (n = 511)
**Maternal characteristics**	
**Maternal age (years), mean (SD)**	30.6 (5.3)
**Ethnicity, n (%)**	
Chinese	240 (47.0%)
Malay	159 (31.1%)
Indian	112 (21.9%)
**Education level, n (%)**	
Secondary or lower	145 (28.6%)
Postsecondary or above	366 (71.4%)
**Family income, n (%)**	
S$0–1999 per month	77 (15.1%)
S$2000–5999 per month	305 (59.7%)
> S$5999 per month	129 (25.2%)
**Infant characteristics**	
**Child's gender, n (%)**	
Male	266 (52.1%)
Female	245 (47.9%)
**Birth order, n (%)**	
First child	217(42.5%)
Second child and above	294(57.5%)

^1^GUSTO, Growing Up in Singapore Towards healthy Outcome

### Dietary intake of the participants of the study

Based on their daily median (IQR) dietary intake, the participants in our cohort had fruit intakes of 88.8 (IQR 50.2–160) g/day, and vegetables of 33.9(IQR 18.2–64.8)g/day. This meant that they met daily recommendations of fruits (80g/day), but only less than half of what is recommended for vegetables (80 g/day)[37,38]. The average intake of wholegrains was only 10.0(IQR 10.0–34.0) g/day, which meant they were also meeting less than half of what is recommended for wholegrains (28g/day) [39]. The median consumption of SSBs was 68.9 (IQR 10–158.8) mL/day, sweet snacks was 45.5(IQR 28.9–75.0)g/day, fast foods was 27.2(IQR 19.2–43.6)g/day and fried foods was 32.7(IQR 21.7–50.3)g/day (Table 2).

**Table 2:** Dietary intake of the study participants in the GUSTO study presented as median and interquartile range (IQR) (n = 511).

Dietary intake	Median(IQR)
Fruit (g/day)	88.8(50.2–160)
Vegetable (g/day)	33.9(18.2–64.8)
Wholegrain (g/day)	10.0(10.0–34.0)
Sugar-sweetened beverages(mL/day)	68.9(10–158.8)
Sweet snacks (g/day)	45.5(28.9–75.0)
Fast foods (g/day)	27.2(19.2–43.6)
Fried foods (g/day)	32.7(21.7–50.3)

### Correlation between the twelve subscales in the CFPQ

The S2 Table shows the correlations amongst the 12 feeding practice subscales. Overall, the subscales showed strengths of correlations ranging from weak to moderate (*r* = 0.00–0.57). Moderate correlations were seen between the modelling healthy food intakes subscale and promoting a healthy environment, encouraging balance and variety, and teaching about nutrition subscales (*r* = 0.42, *r* = 0.47 and *r* = 0.52), and between balance and variety and teaching about nutrition subscales (*r* = 0.57). All other subscales had very weak to weak correlations ranging from *r =* 0.00 to *r* = 0.32.

### Maternal feeding practices associated with children’s fruit, vegetable and wholegrain intakes at 5 years of age

**Fig 1:**
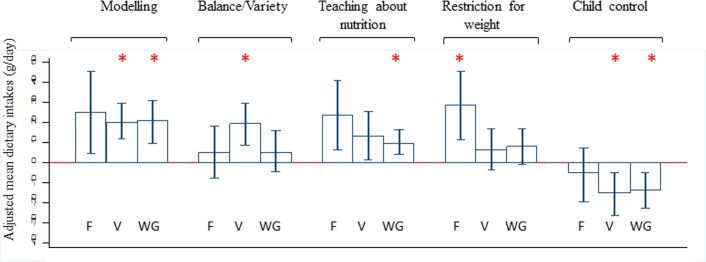
Feeding practices with dietary intakes of fruit(F), vegetable(V) and wholegrain(WG). High feeding practices tertile group versus low tertile as reference group with adjusted mean dietary intakes and error bars representing 95% confidence interval (CI) values. *p<0.006 is statistically significant. ^**1**^Mean intakes were adjusted for confounding variables, such as maternal ethnicity, maternal education level, maternal pregnancy BMI at 15 weeks, child sex, child’s birth order and breastfeeding duration.

Results of the unadjusted and adjusted models were presented in the S3 and S4 Tables, respectively. Only statistically significant findings after the adjustment for potential confounders were represented in a more intuitive way in Fig 1. It shows the adjusted mean differences of fruit, vegetable and wholegrain intake amongst the highest versus lowest tertile (reference category) of five maternal feeding practices (modelling healthy food intakes, encouraging balance and variety in a child’s diet, teaching children about nutrition, food restrictions for weight and allowing child control). The error bars of Fig 1 represent the 95% confidence interval (CI) values.

Mothers in the highest tertile for the practice of modelling healthy food intakes had children who consumed higher amounts of vegetables and wholegrain than those in the lowest tertile (20.0g/day more vegetables and 20.9g/day more wholegrains), but there was no significant differences with fruit intake. Children whose mothers were in the highest tertile for promoting a well-balanced dietary intake and variety is associated with higher intakes of vegetables(19.5g/day more), teaching children about nutrition with higher intakes of wholegrains(9.41g/day more) and restricting foods to control children’s weight with higher intakes of fruits(28.5g/day more), compared to those in the lowest tertile of these feeding practices.

In contrast to these findings, mothers who were in the highest tertile for allowing child control over their food intake (lack of parental control) had children who consumed significantly less vegetables (15.2g/day less) and wholegrains (13.6g/day less).

The other feeding practices such as monitoring unhealthy food intakes, encouraging a healthy environment at home, involving children in food preparation at home, food restrictions for health, pressure to eat, using food as an emotional regulator, and using food as a reward had null associations with fruit, vegetable or wholegrain intake the adjusted models (S4 Table).

### Maternal feeding practices associated with children’s sugar sweetened beverages (SSBs), sweet snacks, fast food and deep fried food at 5 years of age

**Fig 2:**
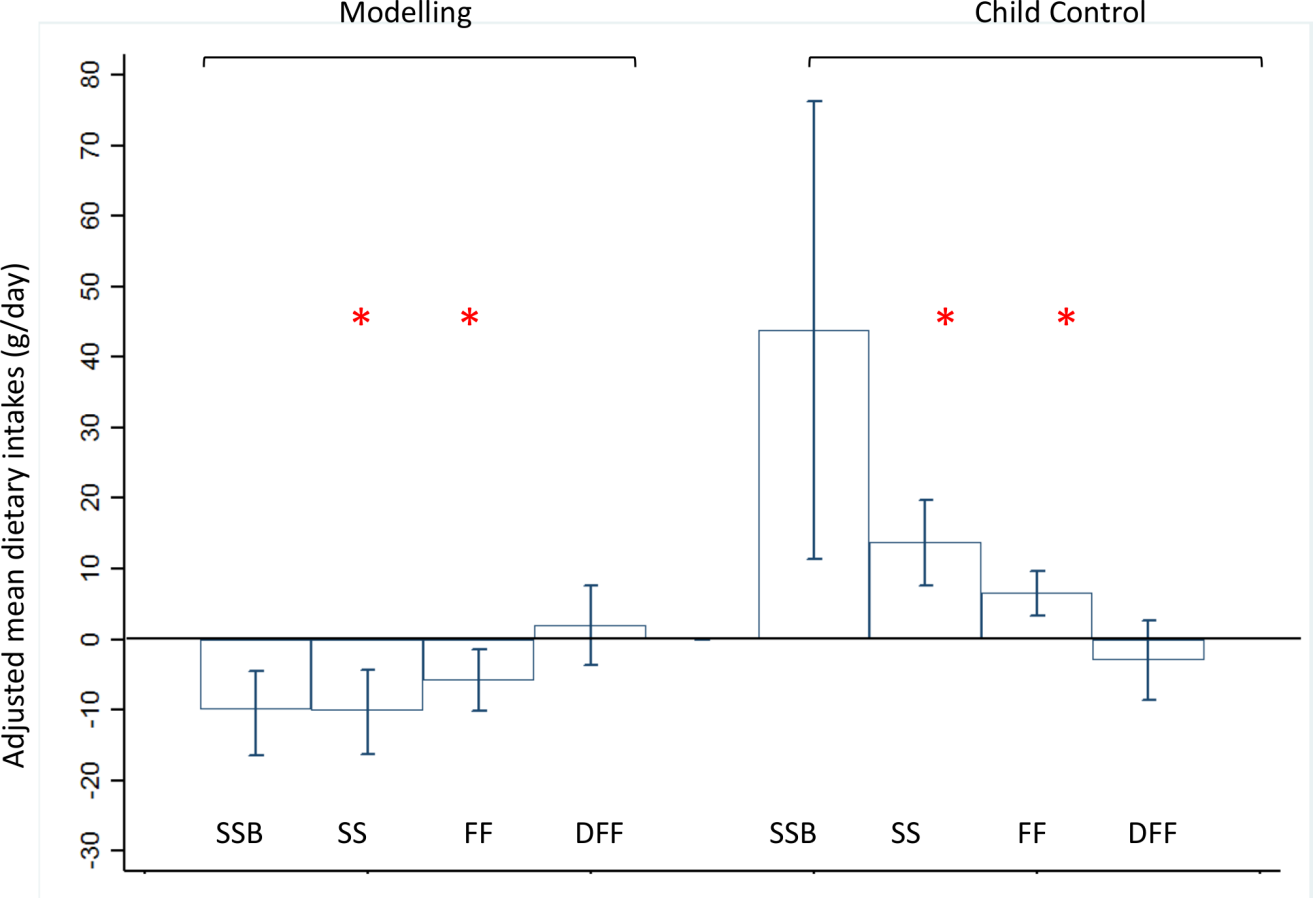
Feeding practices with dietary intakes of sugar sweetened beverages(SSB), sweet snacks(SS), fast foods(FF) and deep fried food(DFF) ^1^. High feeding practices tertile group versus low tertile as reference group with adjusted mean dietary intakes and error bars representing 95% confidence interval (CI) values. *p<0.006 is statistically significant. ^**1**^Mean intakes were adjusted for confounding variables, such as maternal ethnicity, maternal education level, maternal pregnancy BMI at 15 weeks, child sex, child’s birth order and breastfeeding duration.

Results of the unadjusted and adjusted models were presented in S5 and S6 Tables respectively. Again, only statistically significant findings after the adjustment for confounders were presented in Fig 2, which shows the adjusted mean differences of SSBs, sweet snacks, fast foods and deep fried foods amongst the highest versus lowest tertile (reference category) of two maternal feeding practices (modelling healthy food intakes and allowing child control). The error bars of Fig 2 represent the 95% confidence interval (CI) values.

Mothers in the highest tertile for modelling healthy food intakes had children who consumed lower amounts of sweet snacks(10.1g/day less) and fast foods(5.84g/day less). This was approximately a quarter of the median consumption of sweet snacks and fast foods of all the participants of the study (Table 2). In contrast, mothers in the highest tertile for allowing child control had children who consumed higher amounts of sweet snacks (13.7g/day more) and fast foods (6.63g/day more), and again this was approximately a quarter of the median consumption of all the participants of the study (Table 2).

Other feeding practices such as monitoring unhealthy food intakes, encouraging balance and variety, teaching about nutrition, encouraging a healthy environment at home, child involvement in food preparation, and food restrictions for health and weight, pressure to eat, emotional regulation using food, and using food as rewards had null associations with SSBs, sweet snacks, fast foods and deep fried foods in the adjusted models (S6 Table).

### Maternal feeding practices associated with BMI z-scores at 5 years of age

**Fig 3:**
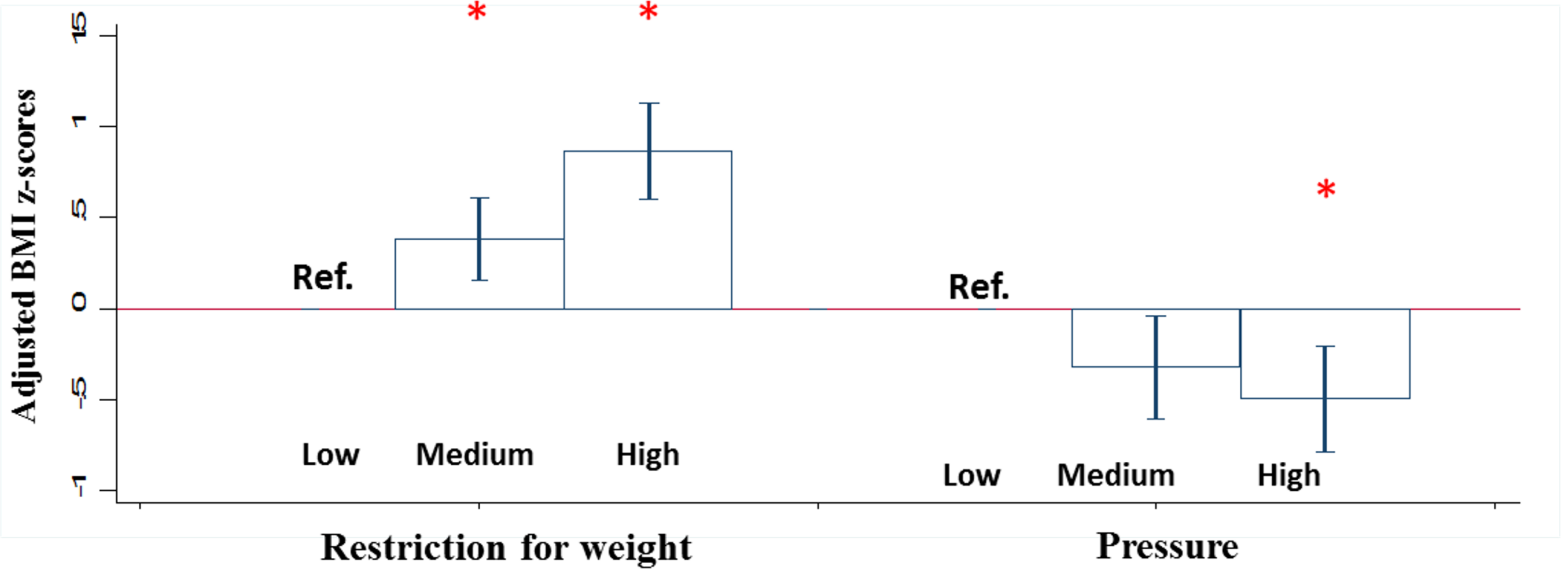
Feeding practices with adjusted BMI z-scores at 5 years of age ^1^. Feeding practices in the high and medium versus low tertile (reference group) with adjusted BMI z-scores at 5 years of age and error bars representing 95% confidence interval (CI) values. *p<0.006 is statistically significant. ^**1**^Mean intakes were adjusted for confounding variables, such as maternal ethnicity, maternal education level, maternal pregnancy BMI at 15 weeks, child sex, child’s birth order and breastfeeding duration.

Fig 3 shows statistically significant associations between maternal feeding practices and BMI *z*-scores at 5 years of age after adjustment for potential confounders, with the error bars representing the 95% confidence interval. Of the 12 feeding practices, only food restrictions for weight and use of pressure to eat were found to be associated with BMI. Mothers in the medium and highest tertile for the practice of food restrictions for weight had children with higher BMI z-scores [β (95%CI): 0.38 SD (95%CI: 0.16, 0.61SD)] and 0.86SD (0.61, 1.21SD) respectively], compared to mothers in the lowest tertile of this practice. Mother’s in the highest tertile for use of pressure to eat had children with BMI z-scores that were half a standard deviation lower than those in the reference group [-0.49 SD (95%CI: -0.78,-0.21SD)]. These significant findings remained with or without adjustment for total energy intake (S7 Table).

## Discussion

In our multi-ethnic Asian Singapore population, we have identified two key feeding practices influencing the dietary intakes of children at ages 5 years. Modelling of healthy food intakes promoted higher intakes of healthier foods such as vegetables and wholegrains, and lower intakes of more discretionary foods such as sweet snacks and fast foods. Conversely, allowing child control led to lower intakes of the same healthy foods, and higher intakes of the same discretionary foods. However, out of the 12 feeding practices studied, only the use of pressure and food restrictions for weight was significantly associated with BMI.

In line with our findings, “modelling” where parents actively demonstrate healthy eating for the child, was previously shown to be associated with vegetable intake [17] and grains[15], but lower intake of sweet snacks and fast foods [15]. Observing a parent consume healthy foods leads to social facilitation of healthier eating habits [40] and food acceptance [41]. It is possible that parents who model eating healthy foods are consuming these foods themselves, making vegetables and wholegrains readily available and accessible, thereby increasing the children’s intakes of these foods.

Encouraging greater balance and variety in a child’s diet was associated with only higher vegetable intake in our study, as reported in previous studies [16,21]. This suggests that mothers in Singapore may interpret promoting variety in the diet as specifically being important for only vegetable intake, but not fruits or wholegrains. Evidence surrounding nutrition education in children, however, is still mixed, studies have reported associations with higher fruit [15,17], but not vegetable intake[16], and lower intake of grains[15].Children of Singaporean mothers who were educated about nutrition had higher wholegrain intakes, possibly because their mothers were focusing on nutritional education at home to promote increased intake of foods that are generally less appealing to them such as wholegrains[42].

Restricting access to foods, especially palatable foods has been shown to be associated with unintended outcomes of higher BMI [17,18,21,43]. In the case of our study, this feeding practice was only associated with higher fruit intakes, but not lower intakes of any discretionary foods [10,11]. Due to the bi-directional nature between this feeding practice and BMI[44], the current findings from our cohort showing the positive association between restriction with higher BMI suggest that mothers are merely reacting to their own perceptions of the child’s weight (of the child being heavier than they actually are)[45], without an actual intention of altering specific dietary intakes of the child[44,46].

The feeding practice of allowing a child control over the foods they select and consume is still not well studied. Studies related specifically to dietary intakes are limited; only one study examined the association between this practice with snacks and simple sugars, and found null associations [15], whereas we showed an association with greater consumption of unhealthy foods, with higher sweet snacks and fast foods. Despite the lack of direct evidence reported on this feeding practice the high use of allowing child control over feeding is characteristic of a permissive parental feeding style [47] which has beenshown to be related to more sugar, fat and junk food consumption in children [48], and greater weight gain [14].

As with other reported studies, the use of pressure to encourage a child to eat was not associated with any of the dietary intakes in our study[17,18], but only with lower BMI *z*-scores [14]. This feeding practice has also been associated with eating behaviours such as lower enjoyment of food [49–51], which has been shown in our own cohort study to be related to lower BMI z-scores [52].This suggests that maternal use of pressure might not have a direct impact on specific foods consumed by the children, but could still influence the overall calorie intake of the child. Like the practice of food restrictions, the use of pressure to eat may be a reaction driven by the mother’s perception that their child is not gaining enough weight[45,53,54], but without the intention of promoting intake or avoidance of specific food groups.

The other six feeding practices, which are encouraging a healthy environment at home, involving a child in food preparation, food restrictions to control a child’s health, monitoring a child’s unhealthy food intake, using food as reward and using food as an emotional regulator showed no associations with dietary intakes or BMI z-scores in this study. A meta-analysis study has shown how the associations between these feeding practices with dietary intakes are mixed, with many studies observing similar null associations [55]. This study revealed that the efficacy of some parenting practices might be dependent on the age of the child. For example, restrictive guidelines and active guidance on nutrition might be less effective in young children because they are less able to follow rules due to limited self-regulation capabilities[55]. Furthermore, studies have reported the practice of using food as reward, and food for emotional regulation relating to subsequent development of emotional eating in children [56,57]. This also suggests that it is possible for some these feeding practices to not have a direct impact on dietary intake in children, but might instead induce emotional eating later on in life.

### Strengths and limitations

Our results further strengthen the evidence that feeding practices can potentially influence children’s dietary intakes and weight status, and suggests that the feeding interactions of parents and their children should be targeted for intervention in Singapore. The strength of the study lies in the large multi-ethnic cohort, the robust multivariate analyses adjusting potential confounders, while adding to the research field by examining a wide range of subscales in the CFPQ. Furthermore, our dietary intake was captured using a quantitative interviewer administered FFQ which is a good representation of habitual intake of children at age 5 years. In addition, we analyzed BMI z-scores with dietary intakes as outcomes of this study which enables us to compare if feeding practices associated with dietary intakes might also be associated with BMI.

However, there are limitations to our study that should be considered: Firstly, this study is cross-sectional, and there’s a possibility of reverse-causation that might bias the interpretation our results. Secondly, both our maternal feeding practices and dietary intakes were based on self-reported questionnaires that are subjected to information bias as some mothers may choose to report more socially desirable dietary intakes and feeding practices. However, the risk of misclassifying our maternal feeding practices is low because the subscales of the CFPQ have previously been validated using external validation, and since this questionnaire was self-administered, we were also more likely to attain valid reports and less socially desirable answers in general [58]. Differential misclassifications might arise from mothers choosing to report socially desirable feeding practices, creating a systematic form of bias. This form of misclassification might then influence the estimates in our study in either direction that could be higher or lower than what the true estimates should be [59].

Our third limitation lies in the generalizability of our study: Our cohort consists of mainly highly educated mothers, and we focused only on preschoolers aged 5, thus the results from this particular study might not be generalizable to the entire Singapore population, especially to older children or other types of caregivers. Lastly, although we have considered many confounders and covariates in our analysis, residual confounding may still remain. In the future, this study could be replicated prospectively to explore long term casual relationships between parental feeding practices, child dietary intake and weight, and it would also benefit from the inclusion of more objective measures of maternal feeding practices and child’s dietary intake.

### Implications for research and practice

In conclusion, our results show for the first time among Asian mother-child pairs, the relationship between maternal modelling of healthy food intakes and allowing child control (lack of parental control) with child dietary intakes, and food restrictions and the use of pressure with BMI z- scores. We saw increased intake of fruits and vegetables by at least a quarter (20g/day) and an increase in wholegrain intake (10g/day) by half of the daily recommend amount, which brings the children in our cohort close to their recommended daily intakes[60]. Our findings provide an empirical basis for recommendations on appropriate feeding practices to help children, especially those who are overweight or those with eating problems to achieve their daily recommended intake of healthy foods. An overall improvement of diet from a combination beneficial of feeding practices might then lead to healthier weight outcomes in children. Furthermore, our estimates in the associations between the use of restriction and pressure with BMI z-scores are within the clinically relevant range for influencing cardiovascular risk factors in overweight children [61]. We believe that feeding practices leading to better diet quality are not always necessarily the same practices associated with lower BMI z-scores in children, suggesting that depending on the health outcomes, certain maternal feeding practices should be prioritized for intervention.

## Supporting information

S1 Table Baseline characteristics of responders and non-responders in the GUSTO study. (DOCX)

S2 Table Pearson’s correlations amongst the twelve maternal feeding practices from the Comprehensive Feeding Practices Questionnaire (CFPQ). (DOCX)

S3 Table Unadjusted mean differences of fruit intake (g/day), vegetable intake (g/day), and wholegrain (g/day) intake across categories of high, medium and low scores of maternal feeding practices at 5 years of age. (DOCX)

S4 Table Multivariate adjusted mean differences of fruit intake (g/day), vegetable intake (g/day), and wholegrain (g/day) intake across tertile categories of high, medium and low scores of maternal feeding practices at 5 years of age. (DOCX)

S5 Table Unadjusted mean differences of sugar-sweetened beverages (SSBs) (mL/day), sweet snacks (g/day), fast-foods (g/day) and fried foods intake (g/day) across tertile categories of high, medium and low scores of maternal feeding practices at 5 years of age. (DOCX)

S6 Table Multivariate adjusted mean differences of sugar-sweetened beverages (SSBs) (mL/day), sweet snacks (g/day), fast-foods (g/day), and fried food intake (g/day) across tertile categories of high, medium and low scores of maternal feeding practices at 5 years of age. (DOCX)

S7 Table Multivariate linear regression of maternal feeding practices across tertile categories of high, medium and low scores with BMI z-scores at 5 years of age ^1^. (DOCX)

S1 Fig Directed acyclic graph–diagram with maternal feeding practices as the exposure and food intake as an outcome. (PNG)

## Abbreviations

CFPQ: Comprehensive Feeding Practices Questionnaire
FFQ: Food Frequency Questionnaire
SSB: Sugar sweetened beverages
BMI: Body Mass Index
