# Supplementary material for: Maternal feeding practices in relation to dietary intakes and BMI in 5 year-olds in a multi-ethnic Asian population
Source: PLoS One. 2018 Sep 18;13(9):e0203045. doi: 10.1371/journal.pone.0203045 (PMC6143183; doi:10.1371/journal.pone.0203045)
Supplement: S1 Table — (DOCX) [file pone.0203045.s001.docx]

Supplementary Table 1: Baseline characteristics of responders and non-responders in the GUSTO study

|  | Responders (n=511) | Non-responders (n=726) | p-value |
| --- | --- | --- | --- |
| **Maternal characteristics** |  |  |  |
| **Maternal age (SD)** | 30.58 (5.27) | 30.58 (5.09) | 0.992 |
| **Ethnicity, n (%)** |  |  | 0.001 |
| Chinese | 240 (47.0%) | 451 (62.1%) |  |
| Malay | 159 (31.1%) | 163 (22.5%) |  |
| Indian | 112 (21.9%) | 112 (15.4%) |  |
| **Education level, n (%)** |  |  | 0.368 |
| Secondary or lower | 145 (28.6%) | 221 (31.0%) |  |
| Postsecondary or above | 362 (71.4%) | 492 (69.0%) |  |
| **Family income, n (%)** |  |  | 0.499 |
| S$0 - 1999 per month | 77 (16.0%) | 104 (15.5%) |  |
| S$2000 - 5999 per month | 274 (57.1%) | 366 (54.5%) |  |
| > S$5999 per month | 129 (26.9%) | 202 (30.1%) |  |
| **Infant characteristics** |  |  |  |
| **Child's gender, n (%)** |  |  | 0.627 |
| Male | 266 (52.1%) | 353 (53.5%) |  |
| Female | 245 (47.9%) | 307 (46.5%) |  |
| **Birth order, n (%)** |  |  | 0.051 |
| First child | 217 (42.5%) | 318 (48.2%) |  |
| Second child and above | 294 (57.5%) | 342 (51.8%) |  |
