# Supplementary material for: Maternal feeding practices in relation to dietary intakes and BMI in 5 year-olds in a multi-ethnic Asian population
Source: PLoS One. 2018 Sep 18;13(9):e0203045. doi: 10.1371/journal.pone.0203045 (PMC6143183; doi:10.1371/journal.pone.0203045)
Supplement: S2 Table — (DOCX) [file pone.0203045.s002.docx]

Supplementary Table 2: Correlations among the twelve CFPQ subscales

|  | Modeling | Balance/variety | Healthy environment | Teaching about nutrition | Involvement | Monitoring | Restriction for weight | Restriction for health | Pressure | Emotional regulation | Child control | Food as reward |
| --- | --- | --- | --- | --- | --- | --- | --- | --- | --- | --- | --- | --- |
| Modeling | 1.00 | 0.47** | 0.42* | 0.52** | 0.30** | 0.26** | 0.18** | 0.32** | 0.18** | -0.07 | -0.18** | 0.04 |
| Balance/variety | - | 1.00 | 0.31** | 0.57** | 0.22** | 0.29** | 0.03 | 0.36** | 0.23** | -0.13** | -0.07 | 0.06 |
| Healthy environment | - | - | 1.00 | 0.32** | 0.23** | 0.24** | 0.17* | 0.17** | -0.02 | -0.16** | 0.31** | -0.18** |
| Teaching about nutrition | - | - | - | 1.00 | 0.29** | 0.24** | -0.00 | 0.30** | 0.08 | -0.14** | 0.14** | -0.03 |
| Involvement | - | - | - | - | 1.00 | 0.06 | 0.17** | 0.06 | 0.05 | 0.03 | -0.01 | 0.19** |
| Monitoring | - | - | - | - | - | 1.00 | 0.10* | 0.24** | -0.00 | -0.09* | -0.20** | 0.00 |
| Restriction for weight | - | - | - | - | - | - | 1.00 | 0.23** | -0.09* | 0.09* | -0.10* | 0.20** |
| Restriction for health | - | - | - | - | - | - | - | 1.00 | 0.21** | -0.00 | 0.13** | 0.22** |
| Pressure | - | - | - | - | - | - | - | - | 1.00 | 0.09* | 0.01 | 0.19** |
| Emotional regulation | - | - | - | - | - | - | - | - | - | 1.00 | 0.23** | 0.30** |
| Child control | - | - | - | - | - | - | - | - | - | - | 1.00 | 0.10* |
| Food as reward | - | - | - | - | - | - | - | - | - | - | - | 1.00 |

* p-value < 0.05; ** p-value < 0.01; r=0.1-0.3 (weak correlation); r=0.3-0.5 (moderate correlation) ; r=>0.5 (strong correlation)
