# Supplementary material for: Maternal feeding practices in relation to dietary intakes and BMI in 5 year-olds in a multi-ethnic Asian population
Source: PLoS One. 2018 Sep 18;13(9):e0203045. doi: 10.1371/journal.pone.0203045 (PMC6143183; doi:10.1371/journal.pone.0203045)
Supplement: S3 Table — (DOCX) [file pone.0203045.s003.docx]

Supplementary Table 3: Unadjusted mean differences of fruit intake (g/day), vegetable intake (g/day), and wholegrain (g/day) intake across categories of high, medium and low scores of maternal feeding practices at 5 years of age.

| **Unadjusted means (95% CI)** | | | |
| --- | --- | --- | --- |
|  | **Total Fruit intake (g/day)** | **Total Vegetable intake (g/day)** | **Total wholegrain intake (g/day)** |
| **Modelling** |  |  |  |
| Low | Reference | Reference | Reference |
| Medium | 19.3 (-3.11, 41.8) | 18.7 (7.03, 30.3)* | 10.5 (0.82, 20.2) |
| High | 49.4 (24.3, 74.6)* | 31.0 (20.6, 41.4)* | 23.5 (10.7, 36.4)* |
| **Balance/variety** |  |  |  |
| Low | Reference | Reference | Reference |
| Medium | 2.48 (-21.5, 26.5) | 5.58 (-6.41, 17.6) | 5.87 (-5.49, 17.2) |
| High | 42.1 (11.9, 72.3)* | 27.0 (12.9, 41.2)* | 16.9 (2.49, 31.2) |
| **Healthy Environment** |  |  |  |
| Low | Reference | Reference | Reference |
| Medium | -1.52(-27.4,24.5) | -1.00(-11.8,11,2) | 10.4(1.89,18.4) |
| High | 36.9(9.88,64.1)* | 23.1(11.6,34.5)* | 27.9(16.9,38.6)* |
| **Teaching about nutrition** |  |  |  |
| Low | Reference | Reference | Reference |
| Medium | 9.76(-16.5,35.6) | 12.5(-0.07,25.4) | 4.05(-6.68,14.8) |
| High | 25.3(2.51,47.8)* | 20.2(10.7,29.7)* | 19.0(-11.6-7.30)* |
| **Involvement** |  |  |  |
| Low | Reference | Reference | Reference |
| Medium | 14.5 (-7.60; 36.6) | -1.98 (-15.7; 11.7) | 5.31 (-9.57; 20.2) |
| High | 41.5 (19.4; 63.4)* | 8.10 (-3.52; 19.7) | 6.00 (-6.07; 18.1) |
| **Monitoring** |  |  |  |
| Low† | Reference | Reference | Reference |
| Medium | -0.16 (-29.1, 28.8) | -6.60 (-18.5, 5.30) | 10.7 (0.75, 20.7) |
| High | 19.3 (-5.75, 44.4) | 10.7 (-0.94, 22.3) | 18.4 (7.74, 29.1)* |
| **Restriction for Weight** |  |  |  |
| Low | Reference | Reference | Reference |
| Medium | 5.43 (-17.7, 28.6) | -2.26 (-13.9, 9.35) | 4.15 (-7.92, 16.2) |
| High | 27.5 (0.29, 54.6) | -0.14 (-11.5, 11.2) | 6.41 (-5.91, 18.7) |
| **Restriction for Health** |  |  |  |
| Low | Reference | Reference | Reference |
| Medium | -1.18 (-24.2, 21.8) | -9.37 (-21.1, 2.40) | -2.53 (-11.5, 6.43) |
| High | 12.3 (-13.3, 37.8) | 2.86 (-9.61,15.3) | 20.2 (6.31, 34.0)* |
| **Pressure** |  |  |  |
| Low | Reference | Reference | Reference |
| Medium | 12.1 (-13.9, 38.2) | 3.30 (-9.68, 16.3) | -1.41 (-15.2, 12.3) |
| High | -1.37 (-23.2, 20.5) | 1.91 (-10.0, 13.8) | -0.60 (-13.8, 12.6) |
| **Emotion Regulation** |  |  |  |
| Low | Reference | Reference | Reference |
| Medium | 9.89 (-15.3, 35.1) | -4.12 (-16.4, 8.12) | -7.83 (-22.3, 6.61) |
| High | 2.82 (-23.3, 28.9) | -11.3 (-23.6, 1.05) | -12.1 (-26.0, 1.75) |
| **Child control** |  |  |  |
| Low | Reference | Reference | Reference |
| Medium | -21.4 (-48.3, 5.45) | -18.0 (-30.6, -5.32) | -7.93 (-23.4,7.52) |
| High | -16.4 (-43.6, 10.8) | -26.6 (-38.4, -14.8)* | -20.7 (-34.9, -6.59)* |
| **Food as Reward** |  |  |  |
| Low | Reference | Reference | Reference |
| Medium | -10.2 (-28.8, 8.53) | -17.1 (-29.0, -5.06) | -9.17 (-23.0,4.69) |
| High | 24.4 (-2.41, 51.2) | -12.5 (-25.2, -0.24) | -6.25 (-20.8, 8.29) |

* p value < 0.004 is statistically significant

The model above present crude unadjusted results
