# Supplementary material for: Maternal feeding practices in relation to dietary intakes and BMI in 5 year-olds in a multi-ethnic Asian population
Source: PLoS One. 2018 Sep 18;13(9):e0203045. doi: 10.1371/journal.pone.0203045 (PMC6143183; doi:10.1371/journal.pone.0203045)
Supplement: S4 Table — (DOCX) [file pone.0203045.s004.docx]

Supplementary Table 4: Multivariate adjusted mean differences of fruit intake (g/day), vegetable intake (g/day), and wholegrain (g/day) intake across tertile categories of high, medium and low scores of maternal feeding practices at 5 years of age.

| **^1^ Adjusted means (95% CI)** | | | |
| --- | --- | --- | --- |
|  | **Total Fruit intake**  **(g/day)** | **Total Vegetable intake (g/day)** | **Total wholegrain intake (g/day)** |
| **Modelling** |  |  |  |
| Low | Reference | Reference | Reference |
| Medium | 17.7 (3.40, 32.8) | 11.4 (-23.87, 24.4) | 12.9 (4.84, 20.0)* |
| High | 25.2 (4.4, 45.4) | 20.0 (11.6, 29.5)* | 20.9 (9.67, 31.1)* |
| **Balance/variety** |  |  |  |
| Low | Reference | Reference | Reference |
| Medium | -9.00 (-24.2, 4.12) | 9.82 (2.17, 17.5) | 2.93 (-4.57,10.4) |
| High | 5.0 (-7.89, 18.1) | 19.5 (8.64, 29.4)* | 5.12 (-4.51, 15.9) |
| **Healthy Environment** |  |  |  |
| Low | Reference | Reference | Reference |
| Medium | 12.2(2.5,23.1) | 10.00 (0.6,20.6) | 7.82(2.32,13.3) |
| High | 12.6 (-1.03,27.6) | 8.00 (-3.8,35.9) | 3.3(-16.7,36.8) |
| **Teaching about nutrition** |  |  |  |
| Low | Reference | Reference | Reference |
| Medium | 11.7 (-10.2, 33.6) | 7.19 (-0.72, 15.1) | -1.80 (-5.52, 23.1) |
| High | 23.5 (6.41, 40.7) | 13.2 (1.50, 25.5) | 9.41(3.98,16.3)* |
| **Involvement** |  |  |  |
| Low | Reference | Reference | Reference |
| Medium | 10.5 (-7.65, 28.6) | 2.08 (-6.03, 10.2) | 1.48 (-8.51, 11.5) |
| High | 12.6 (-1.92, 26.7) | 9.5 (-4.48,19.5) | 4.04 (-5.43, 13.5) |
| **Monitoring** |  |  |  |
| Low | Reference | Reference | Reference |
| Medium | -4.65 (-27.9, 18.6) | -5.46 (-14.1, 3.13) | 4.79 (-5.86, 15.4) |
| High | 11.2 (-8.55, 30.9) | 5.30 (-5.15, 15.8) | 9.30 (-0.87, 19.5) |
| **Restriction for Weight** |  |  |  |
| Low | Reference | Reference | Reference |
| Medium | 8.27 (-8.39, 24.9) | 2.48 (-7.66, 12.6) | 4.99 (-2.19, 12.2) |
| High | 28.5 (11.5, 44.6)* | 6.38 (-3.92, 16.7) | 7.95 (-0.81, 16.7) |
| **Restriction for Health** |  |  |  |
| Low | Reference | Reference | Reference |
| Medium | 6.97 (-12.4, 26.4) | -3.94 (-14.5, 6.65) | -0.54 (-6.75, 5.67) |
| High | 16.3 (-4.68, 37.4) | 4.76 (-7.69,17.2) | 17.5 (4.58, 30.4) |
| **Pressure** |  |  |  |
| Low | Reference | Reference | Reference |
| Medium | 11.5 (-8.13, 31.2) | 7.65 (-1.61, 16.9) | -0.76 (-9.98, 8.47) |
| High | 2.26 (-17.4, 21.9) | 4.89 (-6.33, 16.1) | -1.29 (-10.5, 7.94) |
| **Emotion Regulation** |  |  |  |
| Low | Reference | Reference | Reference |
| Medium | 8.03 (-11.0, 27.0) | -0.01 (-10.9, 10.9) | -7.01 (-18.6, 4.61) |
| High | 9.73 (-13.8, 33.3) | -1.67 (-11.3, 7.93) | -5.41 (-15.7, 4.88) |
| **Child control** |  |  |  |
| Low | Reference | Reference | Reference |
| Medium | -17.6 (-33.2, -3.00) | -12.8 (-23.0, -0.40) | -7.55 (-21.1, 6.02) |
| High | -5.22 (-19.6,7.21) | -15.2 (-26.6, -5.21)* | -13.6 (-22.9, -5.27)* |
| **Food as Reward** |  |  |  |
| Low | Reference | Reference | Reference |
| Medium | -0.58 (-17.3, 16.2) | -12.1 (-22.8, -1.54) | -2.90 (-15.2, 9.43) |
| High | 29.0 (4.91, 53.0) | -0.05 (-13.0, 12.9) | -3.40 (-15.9, 9.10) |

* p-value < 0.006 is statistically significant

^1^The models have been adjusted for maternal ethnicity, maternal education level, maternal pregnancy BMI at 15 weeks , child sex, total energy intake at 5 years, child’s birth order and breastfeeding duration.
