# Supplementary material for: Maternal feeding practices in relation to dietary intakes and BMI in 5 year-olds in a multi-ethnic Asian population
Source: PLoS One. 2018 Sep 18;13(9):e0203045. doi: 10.1371/journal.pone.0203045 (PMC6143183; doi:10.1371/journal.pone.0203045)
Supplement: S5 Table — (DOCX) [file pone.0203045.s005.docx]

Supplementary Table 5: Unadjusted mean differences of sugar-sweetened beverages (SSBs) (mL/day), sweet snacks (g/day), fast-foods (g/day) and fried foods intake (g/day) across categories of high, medium and low scores of maternal feeding practices at 5 years of age.

| **Unadjusted means (95% CI)** | | | | |
| --- | --- | --- | --- | --- |
|  | **Total SSBs intake (mL/day)** | **Total sweet snacks intake (g/day)** | **Total fast-foods intake (g/day)** | **Total fried foods intake (g/day)** |
| **Modelling** |  |  |  |  |
| Low | Reference | Reference | Reference | Reference |
| Medium | -16.2 (-55.7; 23.2) | -15.5 (-27.0; -4.00) | -8.48 (-15.8; -1.14) | -3.57 (-11.3; 4.19) |
| High | -36.0 (-71.5; -0.52) | -15.4 (-27.0; -3.88) | -10.5 (-17.1; -3.80)* | -0.32 (-8.49; 7.84) |
| **Balance/variety** |  |  |  |  |
| Low | Reference | Reference | Reference | Reference |
| Medium | -2.65 (-40.7; 35.4) | 5.61 (-5.54; 16.8) | 0.33 (-7.89; 8.56) | -0.41 (-8.42; 7.60) |
| High | 3.96 (-33.5; 41.4) | -0.74 (-11.8; 10.3) | -3.21 (-11.2; 4.74) | 1.95 (-7.73; 11.6) |
| **Healthy Environment** |  |  |  |  |
| Low | Reference | Reference | Reference | Reference |
| Medium | -15.8(-58.3,26.2) | -18.7(-31.5,-12.5)* | -7.58(-16.4,0.97) | -3.33(-9.87,3.10) |
| High | -60.7(-96.5,-23.9)* | -25.4(-38.4,-12.4)* | -17.9(-25.2,-10.7)* | -2.75(-8.54,5.67) |
| **Teaching about nutrition** |  |  |  |  |
| Low | Reference | Reference | Reference | Reference |
| Medium | -21.7 (-70.2, 26.8) | 2.29 (-10.3, 14.9) | 0.46 (-7.55, 8.47) | 6.38 (-0.37, 13.1) |
| High | -25.9 (-71.1, 19.4) | -1.03 (-13.5, 11.4) | -4.31 (-11.2, 2.62) | 9.34 (1.73, 17.0) |
| **Involvement** |  |  |  |  |
| Low | Reference | Reference | Reference | Reference |
| Medium | -10.1 (-58.8; 38.7) | 4.48 (-7.29; 16.3) | 1.85 (-6.44; 10.1) | 4.54 (-3.93; 13.0) |
| High | -13.7 (-55.8; 28.4) | 5.17 (-5.66; 16.0) | 1.76 (-5.84; 9.36) | 6.38 (-1.61; 14.4) |
| **Monitoring** |  |  |  |  |
| Low | Reference | Reference | Reference | Reference |
| Medium | 6.22 (-29.0; 41.5) | 11.6 (-1.57; 24.7) | 1.43 (-5.00; 7.85) | -0.61 (-9.29; 8.08) |
| High | 4.49 (-28.7; 37.7) | -3.13 (-13.3; 7.06) | 2.26 (-4.01; 8.53) | -0.50 (-9.43; 8.43) |
| **Restriction for Weight** |  |  |  |  |
| Low | Reference | Reference | Reference | Reference |
| Medium | -10.6 (-49.0; 27.8) | 2.15 (-9.30; 13.6) | 2.86 (-3.40; 9.12) | -0.15 (-9.04; 8.75) |
| High | -19.1 (-54.8; 16.6) | -3.77 (-14.8; 7.24) | 3.36 (-3.10; 9.82) | -1.25 (-10.1; 7.59) |
| **Restriction for Health** |  |  |  |  |
| Low | Reference | Reference | Reference | Reference |
| Medium | -2.07 (-41.8; 37.7) | -6.47 (-18.1; 5.15) | -2.13 (-9.17; 4.90) | 1.48 (-6.43; 9.39) |
| High | -32.4 (-65.7; 0.79) | -9.51 (-21.3; 2.28) | -4.50 (-11.7; 2.70) | -1.56 (-10.4; 7.32) |
| **Pressure** |  |  |  |  |
| Low | Reference | Reference | Reference | Reference |
| Medium | -3.83 (-36.88; 29.23) | -0.61 (-11.2; 9.96) | -3.40 (-11.1; 4.34) | -0.67 (-8.59; 7.25) |
| High | 25.80 (-10.35; 61.96) | 1.68 (-9.04; 12.4) | -5.19 (-12.2; 1.80) | 4.21 (-3.62; 12.0) |
| **Emotion Regulation** |  |  |  |  |
| Low | Reference | Reference | Reference | Reference |
| Medium | 15.3 (-18.0; 48.6) | 3.19 (-8.29; 14.7) | -0.12 (-6.51; 6.27) | -3.62 (-12.1; 4.90) |
| High | 37.1 (3.02; 71.3)* | 5.47 (-6.06; 17.0) | 5.26 (-1.21; 11.7) | -0.90 (-10.2; 8.41) |
| **Child control** |  |  |  |  |
| Low | Reference | Reference | Reference | Reference |
| Medium | 46.2 (15.6; 76.7)* | 11.47 (1.10; 21.9) | 6.39 (0.24; 12.6) | -2.02 (-10.9; 6.87) |
| High | 63.3 (33.8; 92.8)* | 24.0 (13.60; 34.3)* | 12.8 (7.39; 18.1)* | -0.52 (-9.68; 8.64) |
| **Food as Reward** |  |  |  |  |
| Low | Reference | Reference | Reference | Reference |
| Medium | -0.35 (-38.4; 37.7) | 5.07 (-5.24; 15.39) | 1.04 (-5.27; 7.36) | 2.80 (-3.68; 9.28) |
| High | -1.00 (-36.9; 34.9) | 13.7 (-5.24; 15.39) | 5.78 (-1.52; 13.1) | 7.32 (-1.56; 16.2) |

* p value < 0.004 is statistically significant

The model above present crude unadjusted results
