# Supplementary material for: Maternal feeding practices in relation to dietary intakes and BMI in 5 year-olds in a multi-ethnic Asian population
Source: PLoS One. 2018 Sep 18;13(9):e0203045. doi: 10.1371/journal.pone.0203045 (PMC6143183; doi:10.1371/journal.pone.0203045)
Supplement: S6 Table — (DOCX) [file pone.0203045.s006.docx]

Supplementary 6: Multivariate adjusted mean differences of sugar-sweetened beverages (SSBs) (mL/day), sweet snacks (g/day), fast-foods (g/day), and fried food intake (g/day) across tertile categories of high, medium and low scores of maternal feeding practices at 5 years of age.

| **^1^ Adjusted mean(95% CI)** | | | | |
| --- | --- | --- | --- | --- |
|  | **Total SSBs intake (mL/day)** | **Total sweet snack intake (g/day)** | **Total fast-foods intake (g/day)** | **Total fried foods intake (g/day)** |
| **Modelling** |  |  |  |  |
| Low | Reference | Reference | Reference | Reference |
| Medium | -5.53 (-40.6, 29.6) | -11.8 (-22.9, -0.68) | -5.55 (-10.3, -0.83) | 0.42 (-5.22, 6.05) |
| High | -10.0 (-16.5, -4.5) | -10.1 (-16.3,-4.94 )* | -5.84 (-10.2, -1.48)* | 2.04 (-3.59, 7.67) |
| **Balance/variety** |  |  |  |  |
| Low | Reference | Reference | Reference | Reference |
| Medium | 18.7 (-11.8, 49.2) | 4.30 (-5.66,14.3) | 1.75 (-3.05, 6.54) | 0.43 (-5.87,6.73) |
| High | 24.9 (-6.44, 56.2) | -8.22 (-15.0, -1.52) | -0.50 (-5.22, 4.22) | 3.00 (-3.73, 9.72) |
| **Healthy Environment** |  |  |  |  |
| Low | Reference | Reference | Reference | Reference |
| Medium | -10.4(-49.7,28.4) | -6.3 (-11.5,-0.8) | -6.09 (-12.8,-0.01) | -0.78(-9.87,3.89) |
| High | -41.2(-80.9,-1.60) | -1.7 (-5.8, 8.30) | -2.54(-1.51,5.59) | -1.45(-8.50,5.12) |
| **Teaching about nutrition** |  |  |  |  |
| Low | Reference | Reference | Reference | Reference |
| Medium | -28.2 (-73.2, 16.8) | 3.29 (-8.02, 14.6) | -0.33 (-5.81, 5.16) | -6.30 (-1.03, 11.6) |
| High | -25.1 (-67.1, 16.8) | -1.89 (-12.1, 8.33) | -3.46 (-8.28, -1.36) | -3.96 (-3.98, -0.15) |
| **Involvement** |  |  |  |  |
| Low | Reference | Reference | Reference | Reference |
| Medium | -8.07 (-49.4, 33.3) | 2.60 (-8.10, 13.2) | 0.91 (-4.14, 5.96) | 4.10 (-2.33, 10.5) |
| High | -8.75 (-47.2, 29.7) | -1.90 (-11.0, 7.21) | -1.41 (-5.32, 2.50) | 2.48 (-2.35, 7.30) |
| **Monitoring** |  |  |  |  |
| Low | Reference | Reference | Reference | Reference |
| Medium | -1.34 (-34.6, 31.9) | 8.49 (-2.97, 20.0) | -1.11 (-6.02, 3.79) | 0.37 (-5.75, 6.48) |
| High | -2.75 (-30.9, 25.4) | -7.11 (-13.2,-1.98) | -0.99 (-5.19, 3.21) | -0.79 (-6.37, 4.79) |
| **Restriction for Weight** |  |  |  |  |
| Low | Reference | Reference | Reference | Reference |
| Medium | -14.0 (-44.4, 16.5) | -1.94 (-12.1, 8.18) | -0.49 (-4.72, 3.73) | -2.85 (-7.95, 2.25) |
| High | -40.1 (-70.3,-9.98) | -9.44 (-19.1, 0.19) | -0.45 (-4.49, 3.59) | -3.04 (-8.55, 2.48) |
| **Restriction for Health** |  |  |  |  |
| Low | Reference | Reference | Reference | Reference |
| Medium | -0.89 (-34.4, 32.6) | -2.62 (-13.2, 7.96) | 0.51 (-4.11, 5.13) | 1.77 (-4.26, 7.79) |
| High | -5.44 (-39.7, 28.8) | -4.10 (-14.8, 6.59) | -1.30 (-5.60, 3.00) | 1.23 (-5.12, 7.58) |
| **Pressure** |  |  |  |  |
| Low | Reference | Reference | Reference | Reference |
| Medium | 16.6 (-9.70, 42.9) | 0.22 (-9.38, 9.81) | 0.94 (-3.62, 5.51) | 1.82 (-3.30, 6.93) |
| High | 41.0 (9.88, 72.1) | 3.06 (-6.39, 12.5) | 1.68 (-2.17, 5.52) | 6.77 (1.09, 12.4) |
| **Emotion Regulation** |  |  |  |  |
| Low | Reference | Reference | Reference | Reference |
| Medium | 20.4 (-1.85, 42.6) | 2.03 (-7.78, 11.8) | 1.04 (-2.73, 4.81) | -1.86 (-7.23, 3.52) |
| High | 15.1 (-51.3, 84.9) | 5.70 (-4.73, 16.1) | 4.79 (0.16, 9.42) | -3.56 (-9.94, 2.82) |
| **Child control** |  |  |  |  |
| Low | Reference | Reference | Reference | Reference |
| Medium | 35.3 (9.95, 60.7) | 7.23 (0.97, 14.9) | 3.96 (0.27, 5.60) | -0.38 (-6.73, 6.00) |
| High | 43.8 (11.4, 76.3) | 13.7 (7.7, 19.8)* | 6.63 (3.55,9.72)* | -2.91 (-8.55, 2.73) |
| **Food as Reward** |  |  |  |  |
| Low | Reference | Reference | Reference | Reference |
| Medium | 2.63 (-30.4, 35.7) | 3.73 (-6.09, 13.6) | 1.80 (-2.12; 5.71) | 0.21 (-4.32; 4.74) |
| High | 1.73 (-27.1, 30.6) | 10.2 (0.71, 19.7) | 4.42 (0.39; 8.45) | 5.32 (-0.86; 11.5) |

* p-value < 0.006 is statistically significant

^1^ The models have been adjusted for maternal ethnicity, maternal education level, maternal pregnancy BMI at 15 weeks, child sex, total energy intake at 5 years, child’s birth order and breastfeeding duration.
