# Supplementary material for: Maternal feeding practices in relation to dietary intakes and BMI in 5 year-olds in a multi-ethnic Asian population
Source: PLoS One. 2018 Sep 18;13(9):e0203045. doi: 10.1371/journal.pone.0203045 (PMC6143183; doi:10.1371/journal.pone.0203045)
Supplement: S7 Table — (DOCX) [file pone.0203045.s007.docx]

Supplementary Table 7: Multivariate linear regression of maternal feeding practices across tertile categories of high, medium and low scores with BMI z-scores at 5 years of age ^1^.

|  | **BMI z-score 5 years** | | | | | | | | | | | | | | | | | | | | | |
| --- | --- | --- | --- | --- | --- | --- | --- | --- | --- | --- | --- | --- | --- | --- | --- | --- | --- | --- | --- | --- | --- | --- |
|  |  | | Model 1 | |  | |  |  | | Model 2 | |  | |  | |  | | | Model 3 | | |  |
|  | β | 95% CI | | | P value | |  | β | 95% CI | | P value | |  | | β | | | 95% CI | | | P value | |
| **Modelling** |  |  | |  | |  | |  |  | |  | |  | |  | |  | | |  | | |
| Low |  | Reference | |  | |  | |  |  | |  | |  | |  | | Reference | | |  | | |
| Medium | 0.02 | -0.29, 0.33 | | 0.921 | |  | | 0.11 | -0.18,0.40 | | 0.465 | |  | | 0.14 | | -0.14,0.43 | | | 0.323 | | |
| High | -0.21 | -0.47, 0.06 | | 0.132 | |  | | -0.13 | -0.39,0.12 | | 0.312 | |  | | -0.13 | | -0.39,0.130 | | | 0.333 | | |
|  |  |  | |  | |  | |  |  | |  | |  | |  | |  | | |  | | |
| **Balance/variety** |  |  | |  | |  | |  |  | |  | |  | |  | |  | | |  | | |
| Low |  | Reference | |  | |  | |  |  | |  | |  | |  | | Reference | | |  | | |
| Medium | -0.09 | -0.41,0.23 | | 0.592 | |  | | -0.08 | -0.27,0.31 | | 0.586 | |  | | -0.09 | | -0.39,0.20 | | | 0.520 | | |
| High | -0.17 | -0.50,0.16 | | 0.303 | |  | | -0.17 | -0.48,0.14 | | 0.300 | |  | | -0.19 | | -0.52,0.13 | | | 0.234 | | |
|  |  |  | |  | |  | |  |  | |  | |  | |  | |  | | |  | | |
| **Healthy environment** |  |  | |  | |  | |  |  | |  | |  | |  | |  | | |  | | |
| Low |  | Reference | |  | |  | |  |  | |  | |  | |  | | Reference | | |  | | |
| Medium | -0.45 | -0.79, -0.11 | | 0.009 | |  | | -0.28 | -0.59,0.02 | | 0.070 | |  | | -0.29 | | -0.59,0.02 | | | 0.068 | | |
| High | -0.55 | -0.89,-0.203 | | 0.002 | |  | | -0.36 | -0.69,-0.04 | | 0.027 | |  | | -0.41 | | -0.74,-0.07 | | | 0.019 | | |
|  |  |  | |  | |  | |  |  | |  | |  | |  | |  | | |  | | |
| **Teaching about nutrition** |  |  | |  | |  | |  |  | |  | |  | |  | |  | | |  | | |
| Low |  | Reference | |  | |  | |  |  | |  | |  | |  | | Reference | | |  | | |
| Medium | 0.21 | -0.10,0.52 | | 0.191 | |  | | 0.15 | -0.14,0.44 | | 0.322 | |  | | 0.12 | | -0.17,0.42 | | | 0.408 | | |
| High | 0.04 | -0.25,0.34 | | 0.772 | |  | | 0.06 | -0.22,0.35 | | 0.656 | |  | | 0.04 | | -0.25,0.33 | | | 0.794 | | |
|  |  |  | |  | |  | |  |  | |  | |  | |  | |  | | |  | | |
| **Involvement** |  |  | |  | |  | |  |  | |  | |  | |  | |  | | |  | | |
| Low |  | Reference | |  | |  | |  |  | |  | |  | |  | | Reference | | |  | | |
| Medium | 0.06 | -0.27,0.39 | | 0.727 | |  | | -0.04 | -0.35,0.26 | | 0.773 | |  | | -.066 | | -0.38,0.25 | | | 0.679 | | |
| High | -0.05 | -0.34, 0.25 | | 0.768 | |  | | -0.11 | -0.40,0.16 | | 0.418 | |  | | -.141 | | -0.43,0.15 | | | 0.346 | | |
|  |  |  | |  | |  | |  |  | |  | |  | |  | |  | | |  | | |
| **Monitoring** |  |  | |  | |  | |  |  | |  | |  | |  | |  | | |  | | |
| Low |  | Reference | |  | |  | |  | Reference | |  | |  | |  | | Reference | | |  | | |
| Medium | 0.28 | -0.02,0.59 | | 0.077 | |  | | 0.20 | -0.08,0.50 | | 0.50 | |  | | 0.171 | | -0.09,0.49 | | | 0.196 | | |
| High | 0.30 | 0.04,0.56 | | 0.028 | |  | | 0.22 | -0.02,0.50 | | 0.50 | |  | | 0.074 | | -0.02,0.47 | | | 0.073 | | |
|  |  |  | |  | |  | |  |  | |  | |  | |  | |  | | |  | | |
| **Restriction for weight** |  |  | |  | |  | |  |  | |  | |  | |  | |  | | |  | | |
| Low |  | Reference | |  | |  | |  | Reference | |  | |  | |  | | Reference | | |  | | |
| Medium | 0.42 | 0.19,0.65 | | 0.001* | |  | | 0.38 | 0.16,0.61 | | 0.001* | |  | | 0.38 | | 0.16,0.61 | | | 0.001* | | |
| High | 0.96 | 0.68,1.23 | | 0.001* | |  | | 0.86 | 0.61,1.21 | | 0.001* | |  | | 0.87 | | 0.60, 1.13 | | | 0.001* | | |
|  |  |  | |  | |  | |  |  | |  | |  | |  | |  | | |  | | |
| **Restriction for health** |  |  | |  | |  | |  |  | |  | |  | |  | |  | | |  | | |
| Low |  | Reference | |  | |  | |  | Reference | |  | |  | |  | | Reference | | |  | | |
| Medium | 0.01 | -0.25,0.28 | | 0.924 | |  | | 0.07 | -0.17,0.32 | | 0.561 | |  | | 0.09 | | -0.16,0.33 | | | 0.509 | | |
| High | 0.19 | -0.09, 0.49 | | 0.195 | |  | | 0.21 | -0.06,0.49 | | 0.129 | |  | | 0.22 | | -0.06,0.49 | | | 0.156 | | |
|  |  |  | |  | |  | |  |  | |  | |  | |  | |  | | |  | | |
| **Pressure** |  |  | |  | |  | |  |  | |  | |  | |  | |  | | |  | | |
| Low |  | Reference | |  | |  | |  |  | |  | |  | |  | | Reference | | |  | | |
| Medium | -0.42 | -0.72,-0.11 | | 0.007 | |  | | -0.32 | -0.61,-0.03 | | 0.029 | |  | | -0.32 | | -0.61,-0.04 | | | 0.037 | | |
| High | -0.64 | -0.94,-0.341 | | 0.001* | |  | | -0.49 | -0.78,-0.21 | | 0.001* | |  | | -0.50 | | -0.79,-0.21 | | | 0.001* | | |
|  |  |  | |  | |  | |  |  | |  | |  | |  | |  | | |  | | |
| **Emotional regulation** |  |  | |  | |  | |  |  | |  | |  | |  | |  | | |  | | |
| Low |  | Reference | |  | |  | |  |  | |  | |  | |  | | Reference | | |  | | |
| Medium | -0.03 | -0.32,0.27 | | 0.878 | |  | | -0.009 | -0.28,0.26 | | 0.944 | |  | | -0.01 | | -0.29, 0.26 | | | 0.931 | | |
| High | -0.22 | -0.53,0.08 | | 0.159 | |  | | -0.24 | -0.53,0.04 | | 0.096 | |  | | -0.24 | | -0.53, 0.04 | | | 0.092 | | |
|  |  |  | |  | |  | |  |  | |  | |  | |  | |  | | |  | | |
| **Child contro**l |  |  | |  | |  | |  |  | |  | |  | |  | |  | | |  | | |
| Low |  | Reference | |  | |  | |  |  | |  | |  | |  | | Reference | | |  | | |
| Medium | -0.10 | -0.41,0.20 | | 0.524 | |  | | -0.09 | -0.38,0.18 | | 0.538 | |  | | -0.09 | | -0.38,0.19 | | | 0.538 | | |
| High | -0.22 | -0.49, 0.05 | | 0.115 | |  | | -0.19 | -0.45,0.06 | | 0.131 | |  | | -0.21 | | -0.46,0.05 | | | 0.116 | | |
|  |  |  | |  | |  | |  |  | |  | |  | |  | |  | | |  | | |
| **Food as reward** |  |  | |  | |  | |  |  | |  | |  | |  | |  | | |  | | |
| Low |  | Reference | |  | |  | |  |  | |  | |  | |  | | Reference | | |  | | |
| Medium | 0.03 | -0.25, 0.32 | | 0.817 | |  | | -0.03 | -0.30,0.24 | | 0.824 | |  | | -0.03 | | -0.29,0.24 | | | 0.851 | | |
| High | -0.08 | -0.39,0.22 | | 0.598 | |  | | -0.09 | -0.30,0.20 | | 0.532 | |  | | -0.11 | | -0.41,0.18 | | | 0.452 | | |

* p-value < 0.006 is statistically significant

^1^ Model 1 is the crude unadjusted model; Model 2 is adjusted for maternal ethnicity, maternal education level, maternal pregnancy BMI at 15 weeks , child sex, child’s birth order and breastfeeding duration; Model 3 is Model 2 with the addition of total energy intake at 5 years as a confounder.
