# Supplementary figures and images for: Maternal feeding practices in relation to dietary intakes and BMI in 5 year-olds in a multi-ethnic Asian population
Source: PLoS One. 2018 Sep 18;13(9):e0203045. doi: 10.1371/journal.pone.0203045 (PMC6143183; doi:10.1371/journal.pone.0203045)

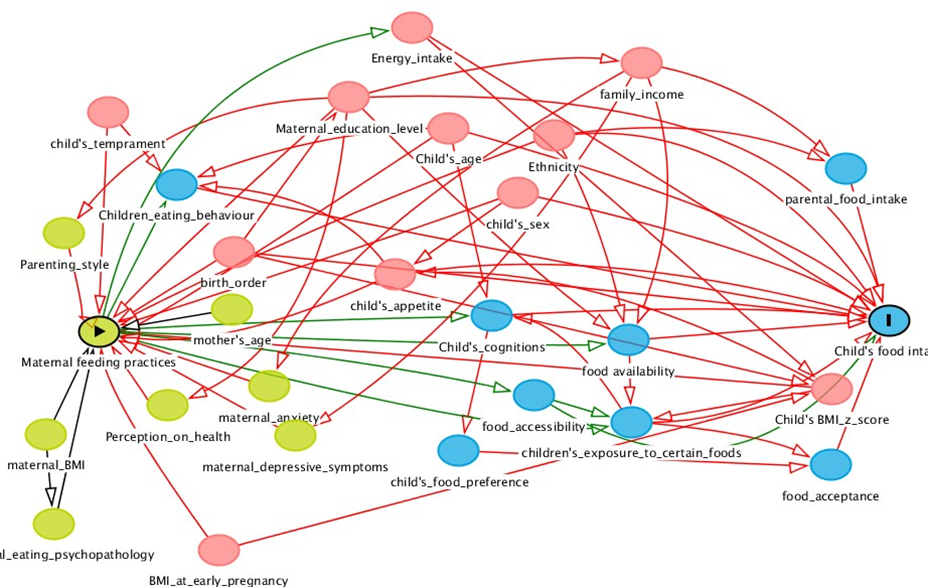

Supplement: S1 Fig — (PNG) [file pone.0203045.s008.png]
